# Extension of emission expectation maximization lookalike algorithms to Bayesian algorithms

**DOI:** 10.1186/s42492-019-0027-4

**Published:** 2019-11-15

**Authors:** Gengsheng L. Zeng, Ya Li

**Affiliations:** 1 Department of Engineering, Utah Valley University, 800 W University Parkway, Orem, UT 84058 USA; 2 Department of Radiology and Imaging Sciences, University of Utah, 729 Arapeen Drive, Salt Lake City, UT 84108 USA; 3 Department of Mathematics, Utah Valley University, 800 W University Parkway, Orem, UT 84058 USA

**Keywords:** Image reconstruction, Tomography, Iterative reconstruction algorithm

## Abstract

We recently developed a family of image reconstruction algorithms that look like the emission maximum-likelihood expectation-maximization (ML-EM) algorithm. In this study, we extend these algorithms to Bayesian algorithms. The family of emission-EM-lookalike algorithms utilizes a multiplicative update scheme. The extension of these algorithms to Bayesian algorithms is achieved by introducing a new simple factor, which contains the Bayesian information. One of the extended algorithms can be applied to emission tomography and another to transmission tomography. Computer simulations are performed and compared with the corresponding un-extended algorithms. The total-variation norm is employed as the Bayesian constraint in the computer simulations. The newly developed algorithms demonstrate a stable performance. A simple Bayesian algorithm can be derived for any noise variance function. The proposed algorithms have properties such as multiplicative updating, non-negativity, faster convergence rates for bright objects, and ease of implementation. Our algorithms are inspired by Green’s one-step-late algorithm. If written in additive-update form, Green’s algorithm has a step size determined by the future image value, which is an undesirable feature that our algorithms do not have.

## Introduction

This work is inspired by Green’s one-step-late (OSL) expectation-maximization (EM) algorithm [1, 2]. Green’s algorithm became popular because it is user-friendly and easy to implement. It has a wide range of applications, such as in positron emission tomography (PET) and single photon emission computed tomography (SPECT) [3–7]. Green’s algorithm also has applications in other fields, such as the minimization of the penalized *I*-divergence [8]. Furthermore, Green’s algorithm may diverge [9]. This study improves Green’s algorithm, making it more stable and more applicable for various noise models.

Green’s algorithm is a maximum a posterior (MAP) algorithm, using image-domain constraints for emission tomography. Other MAP algorithms exist [10–15]. In ref. [12], a method of projection onto convex sets (POCS) was proposed to enforce data fidelity, total-variation (TV) minimization, and image non-negativity. In addition, a GPU algorithm was proposed in ref. [13] to combat the long computation time in combined EM and TV minimization. Filtered backprojection (FBP) reconstruction was proposed for use as the initial image for penalized weighted least-squares (PWLS-TV) reconstruction [12]. Furthermore, in ref. [13] monotonic algorithms for transmission tomography penalized likelihood image reconstruction were developed based on paraboloidal surrogate functions. A similar idea using surrogate functions was reported in refs.[16, 17].

Most recently, we developed a family of emission-EM-lookalike algorithms [10]. These were iterative algorithms in the form of multiplicative image updating, which intrinsically enforced image non-negativity. The unique feature of this family was that the scaling factor was formed by the forward projection of the reconstructed image at the previous iteration, which is a unique feature in the “E-step” in an EM algorithm. Each member of the family had its own noise model. This work will extend this family of emission-EM-lookalike algorithms to Bayesian algorithms, by introducing a new factor. The three main features of the proposed algorithms comprise multiplicative updating with a non-negativity constraint, weighting by a projection noise model, and the incorporation of Bayesian constraints.

Many MAP algorithms in image reconstruction, especially in transmission tomography, employ the POCS methodology, which is an alternating optimization method. This breaks the objective function into different parts and optimizes each part separately. Our proposed method optimizes the objection function with all constraints considered simultaneously.

## Methods

### Modification of iterative Green’s OSL algorithm

We first provide a brief review of Green’s algorithm, before extending it. The iterative Green’s OSL algorithm can be expressed as [1, 2].

1 $$ {x}_{i,j}^{\left(n+1\right)}=\frac{x_{i,j}^{(n)}}{\sum \limits_k{a}_{\left(i,j\right)k}+\beta {U}_{i,j}^{(n)}}\sum \limits_k{a}_{\left(i,j\right)k}\frac{p_k}{\sum \limits_{\hat{i},\hat{j}}{a}_{\left(\hat{i},\hat{j}\right)k}{x}_{\hat{i},\hat{j}}^{(n)}}, $$

where ${x}_{i,j}^{(n)}$ is the reconstructed image pixel (*i, j*) at the *n*th iteration, *p*_*k*_ is the *k*th ray-sum measurement, *a*_(*i,j*)*k*_ is the contribution of the pixel *x*_*i,j*_ to the measurement *p*_*k*_, *β* is a control parameter, and ${U}_{i,j}^{(n)}$ is the derivative of a penalty function *V* with respect to the image pixel ${x}_{i,j}^{(n)}$ at the *n*th iteration, i.e.,

2 $$ {U}_{i,j}^{(n)}=\frac{\partial V}{\partial {x}_{\left(i,j\right)}^{(n)}}. $$

Using the approximation 1/(1+*x*)≈1-*x* when │*x*│<<1, the first factor ${x}_{i,j}^{(n)}/\left[\sum \limits_k{a}_{\left(i,j\right)k}+\beta {U}_{i,j}^{(n)}\right]$ in algorithm (1) can be approximated as $\frac{x_{i,j}^{(n)}}{\sum \limits_k{a}_{\left(i,j\right)k}}\left(1-\frac{\beta }{\sum \limits_k{a}_{\left(i,j\right)k}}{U}_{i,j}^{(n)}\right)$. Here, $\sum \limits_k{a}_{\left(i,j\right)k}$, is in general not a constant. If $\beta /\sum \limits_k{a}_{\left(i,j\right)k}$ is not a constant, then the constraint *U* is not uniformly enforced throughout the image. To improve the algorithm, we simply discard $\sum \limits_k{a}_{\left(i,j\right)k}$ in $\beta /\sum \limits_k{a}_{\left(i,j\right)k}$. Thus, our proposed modification of Green’s algorithm is

3 $$ {x}_{i,j}^{\left(n+1\right)}=\left(1-\beta {U}_{i,j}^{(n)}\right)\frac{x_{i,j}^{(n)}}{\sum \limits_k{a}_{\left(i,j\right)k}}\sum \limits_k{a}_{\left(i,j\right)k}\frac{p_k}{\sum \limits_{\hat{i},\hat{j}}{a}_{\left(\hat{i},\hat{j}\right)k}{x}_{\hat{i},\hat{j}}^{(n)}}. $$

We will gain further insight into this modification by rewriting both the original Green’s algorithm (1) and the modified algorithm (3) in the additive-update form (that is, in the form of gradient descent). The additive form can be expressed as

4 $$ {x}_{i,j}^{\left(n+1\right)}={x}_{i,j}^{(n)}-{\lambda}_1{U}_{i,j}^{(n)}-{\lambda}_2\sum \limits_k{a}_{\left(i,j\right)k}{w}_k\left(\sum \limits_{\hat{i},\hat{j}}{a}_{\left(\hat{i},\hat{j}\right)k}{x}_{\hat{i},\hat{j}}^{(n)}-{p}_k\right), $$

where

5 $$ {w}_k=\frac{1}{\sum \limits_{\hat{i},\hat{j}}{a}_{\left(\hat{i},\hat{j}\right)k}{x}_{\hat{i},\hat{j}}^{(n)}} $$

is the noise-weighting factor for the Poisson noise model and

6 $$ {\lambda}_2=\frac{x_{i,j}^{(n)}}{\sum \limits_k{a}_{\left(i,j\right)k}} $$

is the step size for projection data fidelity minimization. In algorithm (4), λ_1_ is the step size for Bayesian constraint minimization. For the original Green’s algorithm (1),

7 $$ {\lambda}_1^{(original)}=\beta {x}_{i,j}^{\left(n+1\right)}/\sum \limits_k{a}_{\left(i,j\right)k}, $$

whereas for the revised algorithm (3),

8 $$ {\lambda}_1^{(revised)}=\beta \frac{x_{i,j}^{(n)}}{\sum \limits_k{a}_{\left(i,j\right)k}}\sum \limits_k{a}_{\left(i,j\right)k}\frac{p_k}{\sum \limits_{\hat{i},\hat{j}}{a}_{\left(\hat{i},\hat{j}\right)k}{x}_{\hat{i},\hat{j}}^{(n)}}. $$

The most significant difference between algorithms (7) and (8) is that the factor ${\lambda}_1^{(original)}$ in (7) depends on the future image ${x}_{i,j}^{\left(n+1\right)}$, while the factor ${\lambda}_1^{(revised)}$ in (8) depends only on the current image ${x}_{i,j}^{(n)}$.

It is required that the image *x*_*i,j*_ is non-negative. It can be observed from algorithm (3) that if $\beta {U}_{i,j}^{(n)}>1$, then the sign of *x*_*i,j*_ will alternate. Therefore, a necessary condition for the image to be non-negative is $\beta {U}_{i,j}^{(n)}<1$. This intrinsic non-negativity constraint is guaranteed by the requirement that $\beta {U}_{i,j}^{(n)}<1$ if the initial image is positive. This can be readily observed by noticing that every factor in algorithm (3) is non-negative.

One way to prevent this from occurring is to introduce a sigmoid function *φ*, and to replace $\beta {U}_{i,j}^{(n)}$ by $\phi \left(\beta {U}_{i,j}^{(n)}\right)$. There are many ways to define a sigmoid function *φ*. For example, one may choose $\phi (x)=x/\sqrt{1+{x}^2}$.

In deriving the Green’s algorithm using prior information [1], it is necessary to evaluate the derivative of the energy function *V*, which carries the prior information. This energy function is defined for the updated image, which is not yet available. In Green’s algorithm, an approximation is performed to evaluate this derivative of the energy function, using the current image to replace the future image. This approximation is termed “one-step-late”.

The derivation of the EM-lookalike algorithms in ref. [10] was based on the noise variance model, unlike the conventional approach based on a random variable distribution function. Our derivation only considered two items: (1) the noise variance in the projections and (2) the non-negativity constraint for the image.

The derivation of the MAP in this study can been considered as an approximation of Green’s MAP algorithm using 1/(1+*x*)≈1-*x* when │*x*│<<1. The proposed algorithms are in the form of “(1*-βU*) × (EM-lookalike).” When *β* = 0, this form is exactly the EM-lookalike form. The factor (1*-βU*) is new in this work, to minimize a Bayesian function *V* whose gradient is the function *U*. By driving *U* to zero, the Bayesian function *V* is minimized. The additive form algorithm (4) reveals that the proposed algorithms minimize the objective function

9 $$ F=V+\frac{1}{2}\sum \limits_k{w}_k{\left(\sum \limits_{i,j}{a}_{\left(i,j\right)k}{x}_{i,j}-{p}_k\right)}^2, $$

where the functions *U* and *V* are related as

10 $$ {U}_{i,j}^{(n)}=\frac{\partial V}{\partial {x}_{\left(i,j\right)}^{(n)}}. $$

For a different noise model, we can simply change the noise weighting *w*_*k*_ as in ref. [10].

This study builds on ref. [10], by considering a new energy function *V* and forcing its gradient *U* to zero. This point can be intuitively appreciated from the additive form algorithm (4).

From algorithm (6), we observe that the ML-EM algorithm’s step size *λ*_2_ is scaled by the image pixel value ${x}_{i,j}^{(n)}$ at the *n*th iteration. As a result, brighter objects converge faster than darker objects.

From algorithm (5), we observe that the weighting factor *w*_*k*_ is the reciprocal of the estimated mean value of the *k*th ray-sum at the *n*th iteration. Note that *w*_*k*_ will change with different noise models.

From algorithm (7), we observe that *λ*_1_ depends on the image value of the next iteration. This feature is undesirable, because it may cause the algorithm diverge. This undesirable feature has been removed from the revised algorithm, as shown in algorithm (8), where *λ*_1_ depends only on the current image value.

The parameter *λ*_2_ is intrinsically determined by the conventional ML-EM algorithm. The parameter *λ*_1_ is affected by the parameter *β*. For any penalty function *V*, the parameter *β* is chosen by trial-and-error. When in doubt, a smaller positive *β* value should be chosen.

If the true solution with ${\sum}_{\hat{i},\hat{j}}{a}_{\left(\hat{i},\hat{j}\right)k}{x}_{\hat{i},\hat{j}}={p}_k$ and *U*_*i,j*_ *=* 0 exits, then it is straightforward to verify that the true solution is a fixed point of the proposed algorithm (3). In fact, letting ${\sum}_{\hat{i},\hat{j}}{a}_{\left(\hat{i},\hat{j}\right)k}{x}_{\hat{i},\hat{j}}^{(n)}={p}_k$ and ${U}_{i,j}^{(n)}=0$, the right-hand side of (3) becomes ${x}_{i,j}^{(n)}$.

### Modified algorithm for no weighting

We now consider a hypothetical imaging system, where the noise in the measurements is identically distributed with the same variance. In this case, noise weighting should not be utilized in the image reconstruction algorithm. The ML-EM lookalike algorithm for this hypothetical case is given as [10].

11 $$ {x}_{i,j}^{\left(n+1\right)}={x}_{i,j}^{(n)}\frac{\sum \limits_k{a}_{\left(i,j\right)k}{p}_k}{\sum \limits_k{a}_{\left(i,j\right)k}\sum \limits_{\hat{i},\hat{j}}{a}_{\left(\hat{i},\hat{j}\right)k}{x}_{\hat{i},\hat{j}}^{(n)}}. $$

Using our strategy of introducing a simple new factor $\left(1-\beta {U}_{i,j}^{(n)}\right)$, the Bayesian algorithm associated with algorithm (11) is proposed as

12 $$ {x}_{i,j}^{\left(n+1\right)}=\left(1-\beta {U}_{i,j}^{(n)}\right){x}_{i,j}^{(n)}\frac{\sum \limits_k{a}_{\left(i,j\right)k}{p}_k}{\sum \limits_k{a}_{\left(i,j\right)k}\sum \limits_{\hat{i},\hat{j}}{a}_{\left(\hat{i},\hat{j}\right)k}{x}_{\hat{i},\hat{j}}^{(n)}}. $$

### Modified algorithm for the transmission noise model

The variance of the transmission tomography sinogram is proportional to the exponential function of the sinogram’s mean value [11]:

13 $$ \mathit{\operatorname{var}}\left({p}_k\right)\propto \exp \left({\overline{p}}_k\right)\approx \exp \left(\sum \limits_{\hat{i},\hat{j}}{a}_{\left(\hat{i},\hat{j}\right)k}{x}_{\hat{i},\hat{j}}^{(n)}\right). $$

An ML-EM lookalike algorithm for the transmission data is derived in ref. [10] as

14 $$ {x}_{i,j}^{\left(n+1\right)}={x}_{i,j}^{(n)}\frac{\sum \limits_k\left[{a}_{\left(i,j\right)k}{p}_k\exp \left(-\sum \limits_{\hat{i},\hat{j}}{a}_{\left(\hat{i},\hat{j}\right)k}{x}_{\hat{i},\hat{j}}^{(n)}\right)\right]}{\sum \limits_k\left[{a}_{\left(i,j\right)k}\left(\sum \limits_{\hat{i},\hat{j}}{a}_{\left(\hat{i},\hat{j}\right)k}{x}_{\hat{i},\hat{j}}^{(n)}\right)\exp \left(-\sum \limits_{\hat{i},\hat{j}}{a}_{\left(\hat{i},\hat{j}\right)k}{x}_{\hat{i},\hat{j}}^{(n)}\right)\right]}. $$

It is straightforward to modify algorithm (14) to a Bayesian algorithm, by introducing a new factor $\left(1-\beta {U}_{i,j}^{(n)}\right)$ as follows:

15 $$ {\displaystyle \begin{array}{l}{x}_{i,j}^{\left(n+1\right)}=\left(1-\beta {U}_{i,j}^{(n)}\right){x}_{i,j}^{(n)}\\ {}\times \frac{\sum \limits_k\left[{a}_{\left(i,j\right)k}{p}_k\exp \left(-\sum \limits_{\hat{i},\hat{j}}{a}_{\left(\hat{i},\hat{j}\right)k}{x}_{\hat{i},\hat{j}}^{(n)}\right)\right]}{\sum \limits_k\left[{a}_{\left(i,j\right)k}\left(\sum \limits_{\hat{i},\hat{j}}{a}_{\left(\hat{i},\hat{j}\right)k}{x}_{\hat{i},\hat{j}}^{(n)}\right)\exp \left(-\sum \limits_{\hat{i},\hat{j}}{a}_{\left(\hat{i},\hat{j}\right)k}{x}_{\hat{i},\hat{j}}^{(n)}\right)\right]}.\end{array}} $$

In general, a Bayesian algorithm can be readily obtained from a multiplicative-update image reconstruction algorithm by introducing a new factor $\left(1-\beta {U}_{i,j}^{(n)}\right)$. The resulting Bayesian algorithm remains multiplicative.

### The TV penalty function

Any penalty function *V* can be employed in the proposed algorithm (3). Some constraints encourage smoothing, such as the maximum entropy constraint [18], because their main goal is denoising. Maximum entropy algorithms tend to over-smooth images, and as a result sharp edges are not maintained. Thus, maximum entropy algorithms are not popular for CT image reconstruction. On the other hand, TV-type constraints can reduce noise and maintain sharp edges when the parameters are suitably chosen. Here, we select the TV norm for a feasibility evaluation:

16 $$ V=\sum \limits_{i,j}\sqrt{{\left({x}_{i,j}-{x}_{i,j+1}\right)}^2+{\left({x}_{i,j}-{x}_{i+1,j}\right)}^2}, $$

where *x*_*i,j*_ is a pixel value in a two-dimensional (2D) image. The associated derivative (2) is given as

17 $$ {\displaystyle \begin{array}{l}{U}_{i,j}\approx \frac{\left({x}_{i,j}-{x}_{i,j+1}\right)+\left({x}_{i,j}-{x}_{i+1,j}\right)}{\sqrt{{\left({x}_{i,j}-{x}_{i,j+1}\right)}^2+{\left({x}_{i,j}-{x}_{i+1,j}\right)}^2+\varepsilon }}\\ {}+\frac{x_{i,j}-{x}_{i,j-1}}{\sqrt{{\left({x}_{i,j-1}-{x}_{i,j}\right)}^2+{\left({x}_{i,j-1}-{x}_{i+1,j-1}\right)}^2+\varepsilon }}\\ {}+\frac{x_{i,j}-{x}_{i-1,j}}{\sqrt{{\left({x}_{i-1,j}-{x}_{i-1,j+1}\right)}^2+{\left({x}_{i-1,j}-{x}_{i,j}\right)}^2+\varepsilon }}.\end{array}} $$

Here, the small value of *ε* is introduced to prevent the denominator being zero. In this study, *ε* = 0.0001 is adopted.

### Computer simulations

Two sets of computer simulations were conducted, using emission and transmission noise models, respectively. The simulation setup for the emission data is as follows.

There were 180 projection views over 360°. The images were reconstructed in an array of size 128 × 128 (pixels). A parallel-hole collimation was assumed for the data generation. The detector had 128 detection bins, and the bin size was the same as the image pixel size.

**Fig. 1:**
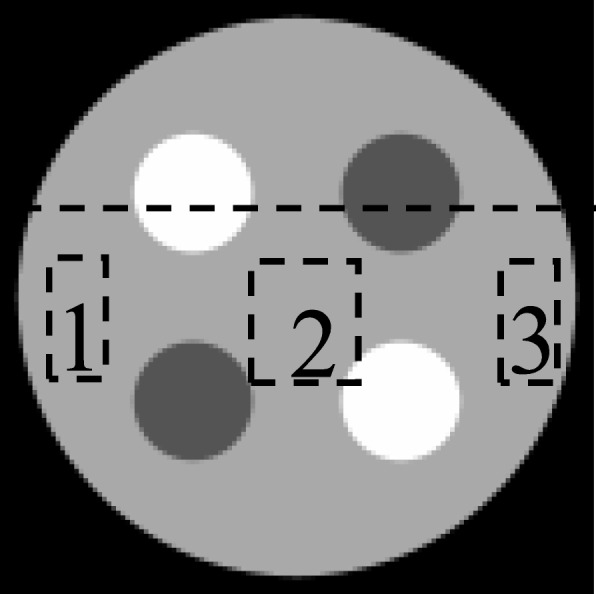
Computer simulated phantom. The dotted horizontal line indicates the location of line-profiles. Regions 1, 2, and 3 were used for TV-norm evaluation

A 2D circular phantom with a diameter of 120.32 pixels was employed in the simulations. The phantom, based on SPECT imaging, contained two small cold disks and two small hot disks, all with a diameter of 25.6 pixels, as shown in Fig. 1. The image intensity of the large circular disk was defined as 1 unit. The cold disks had an intensity value of 0.5, and the hot disks had an intensity value of 1.5. The projections were generated analytically, without using discrete pixels, and noisy projections were generated using the Poisson noise model. The total number of counts was approximately 2 × 10^6^.

The computer simulation setup for the transmission data was as follows. A parallel-beam imaging geometry was assumed. The image array was of size 512 × 512, the number of views was 400 over 180°, and the number of detection channels was 512. The transmission phantom looked similar to the emission phantom (Fig. 1), except four times larger. The pixel length was 0.5 mm. Furthermore, the attenuation coefficient was 0.0193 mm^− 1^ for the large disc, 0.0269 mm^− 1^ for the small circular bright regions, and 0.0083 mm^− 1^ for the small circular dark regions.

The transmission CT noise model was adopted for the sinogram data with very low counts, where the sinogram variance was proportional to the exponential function of the sinogram value. Two x-ray influxes were considered: *I*_0_ = 100 and *I*_0_ = 10,000.

Three regions were selected in the image for TV-norm noise evaluation. Note that the TV norm can measure the image fluctuation. These regions are depicted in Fig. 1. The average of the TV norms in these regions was employed as a figure-of-merit for noise evaluation. Furthermore, a line profile was provided for each reconstructed image. The location of the line profile is indicated in Fig. 1. As an additional figure-of-merit, the mean-squared-error (MSE) was also calculated between the reconstruction and true profiles, and this is reported in the figures.

## Results

### Emission data simulation results

**Fig. 2:**
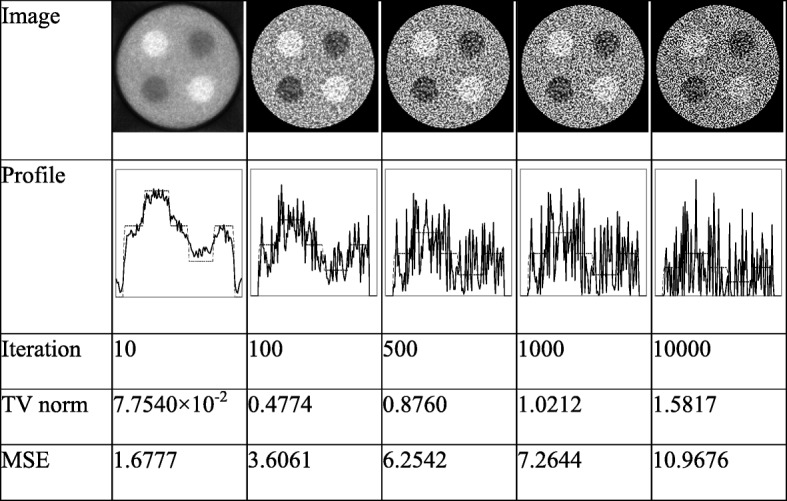
Reconstructions of emission data using the conventional ML-EM algorithm

**Fig. 3:**
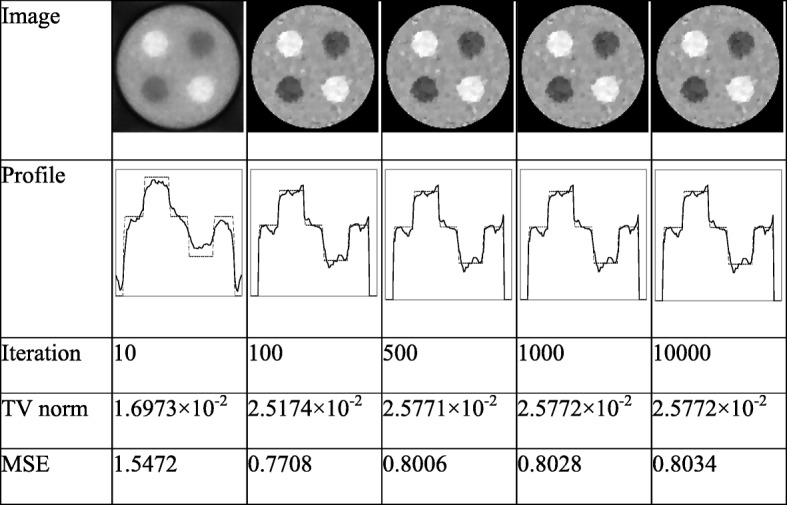
Reconstructions of emission data using Green’s OSL algorithm with *β* = 1.2

**Fig. 4:**
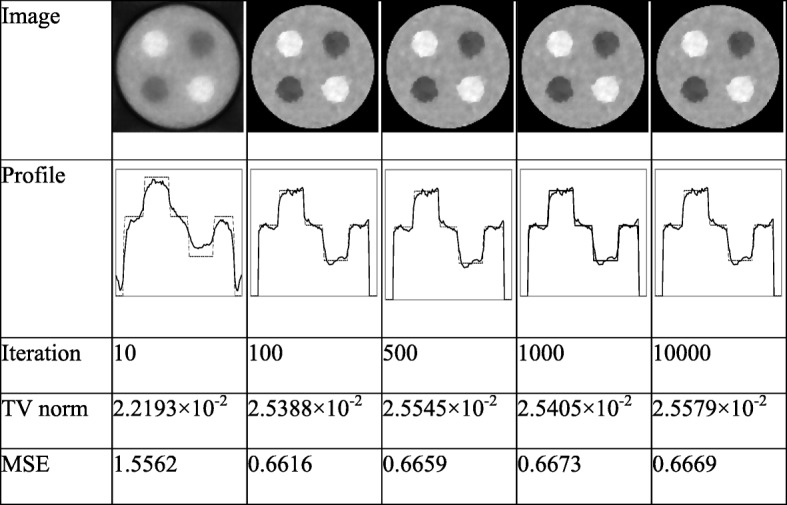
Reconstructions of emission data using the proposed algorithm with *β* = 0.01

Three algorithms were used to reconstruct the images: the conventional ML-EM algorithm (by setting *β* = 0 in either (1) or (9)), Green’s OSL algorithm (1), and the proposed algorithm (9). The results are depicted in Figs. 2, 3, and 4, respectively, for the three algorithms. The proposed algorithm and Green’s OSL algorithm yield similar performances.

The parameter *β* in the revised algorithm (3) is approximately equal to *β* in the original Green’s algorithm (1) divided by the backprojection value of the constant 1. Roughly speaking, the *β* value in the original Green’s algorithm is the *β* value in the revised algorithm times the number of view angles. In our example, *β* = 1.2 for the original Green’s algorithm and *β* = 0.01 for the revised algorithm, and the number of view angles is 180. Thus, the regularization in Fig. 4 is a little stronger than that in Fig. 3.

### Transmission data simulation results

**Fig. 5:**
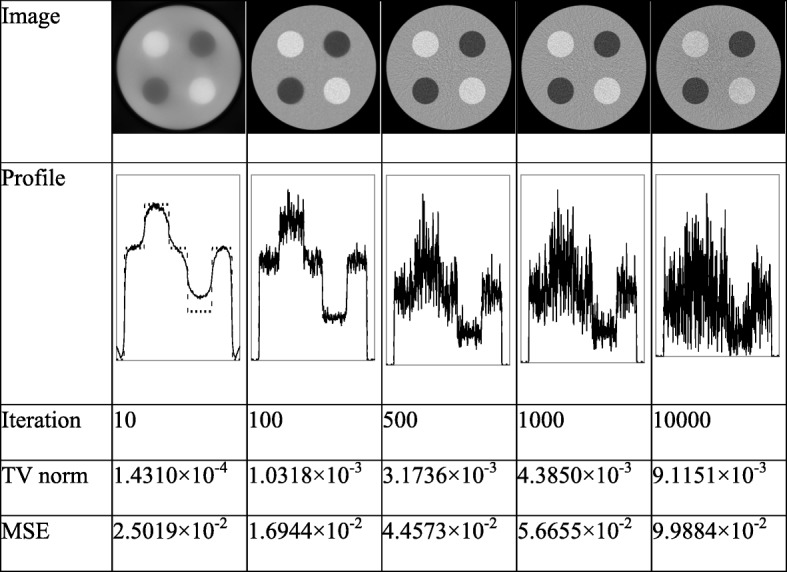
Reconstructions of transmission data using algorithm (14) when *I*_0_ = 10,000

**Fig. 6:**
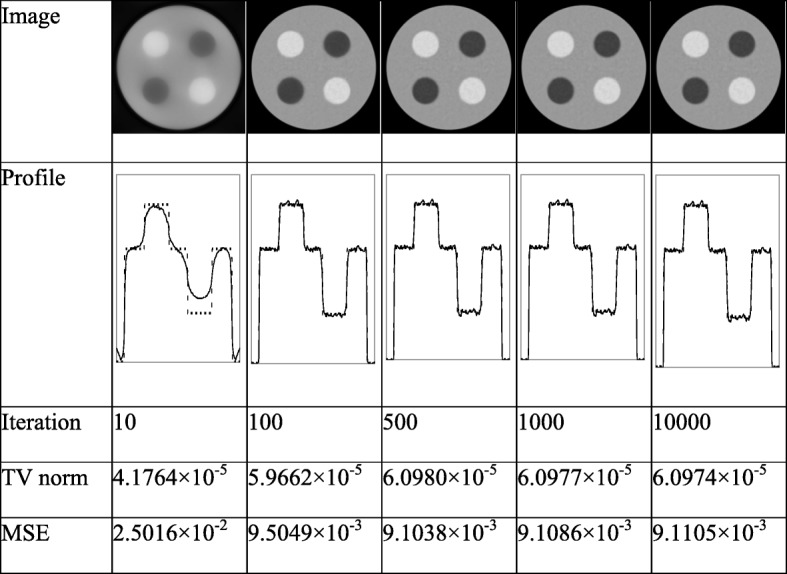
Reconstructions of transmission data using algorithm (15) with *β* = 0.01 when *I*_0_ = 10,000

**Fig. 7:**
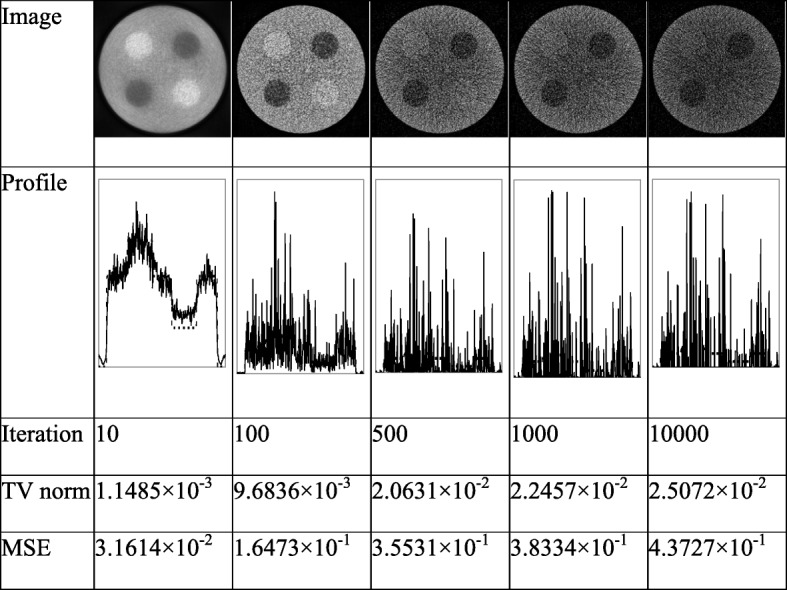
Reconstructions of transmission data using algorithm (14) when *I*_0_ = 100

**Fig. 8:**
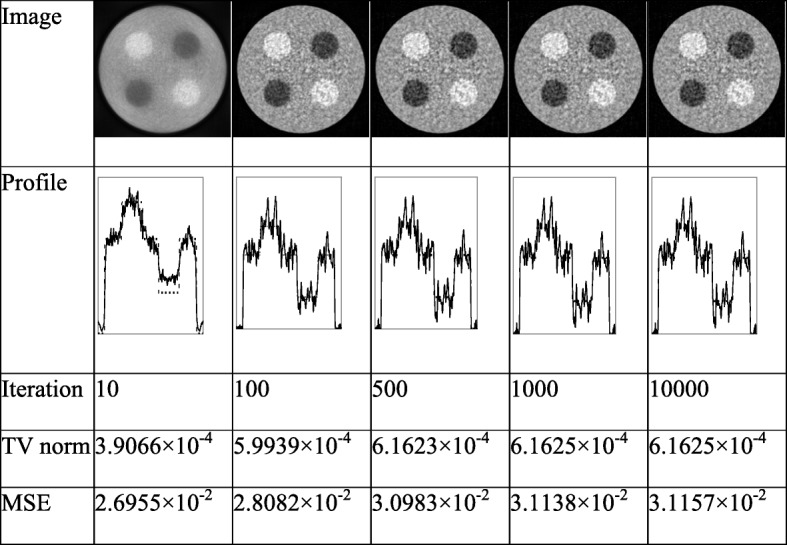
Reconstructions of transmission data using algorithm (15) with *β* = 0.01 when *I*_0_ = 100

Two algorithms were used to reconstruct the images: the EM-lookalike transmission algorithm (14) and proposed algorithm (15). For each algorithm, images were reconstructed with two noise levels. The results are presented in Figs. 5, 6, 7, and 8, for the two algorithms and two noise levels.

**Fig. 9:**
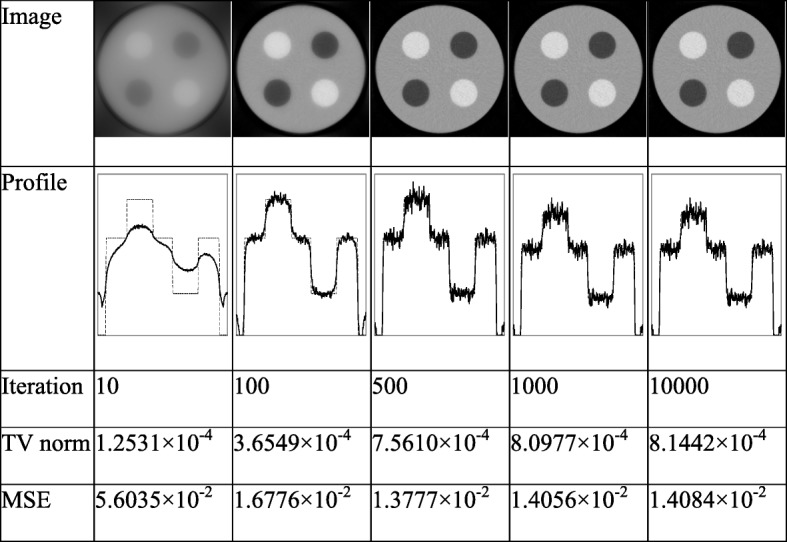
Reconstructions with transmission data using the POCS algorithm in ref. [12] when *I*_0_ = 10,000

**Fig. 10:**
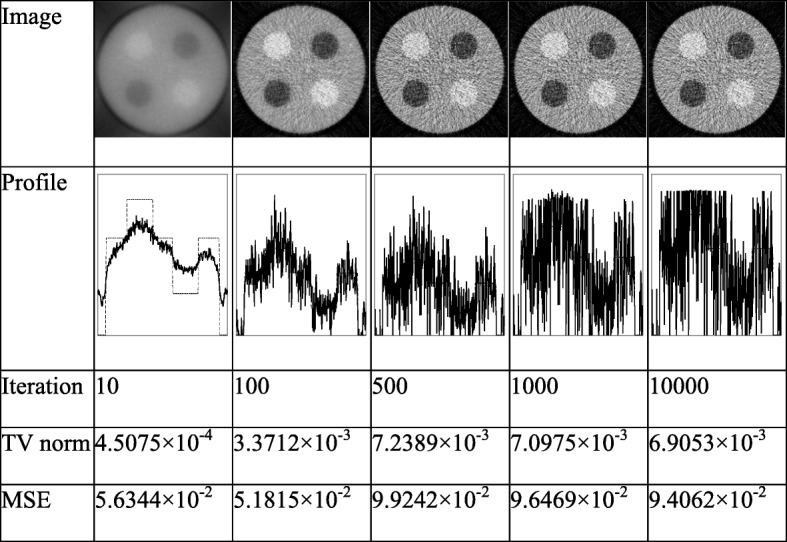
Reconstructions with transmission data using the POCS algorithm in ref. [12] when *I*_0_ = 100

Finally, for comparison purposes we implemented the POCS algorithm proposed in ref. [12] and used it to reconstruct the transmission images. The results are presented in Figs. 9 and 10, for the lower and higher noise cases, respectively. We observe that our proposed simultaneous optimization algorithm performs better than the POCS algorithm proposed in ref. [12] in this task, in terms of the TV norm and MSE results.

It can be observed that the central region of the phantom appears darker in the Fig. 7. We hypothesize that noise may affect the convergence rate in an iterative algorithm. If a system of linear equations is more consistent, then the convergence rate may be faster. If the data is noisier and the system is less consistent, then the convergence rate may be slower.

**Fig. 11:**
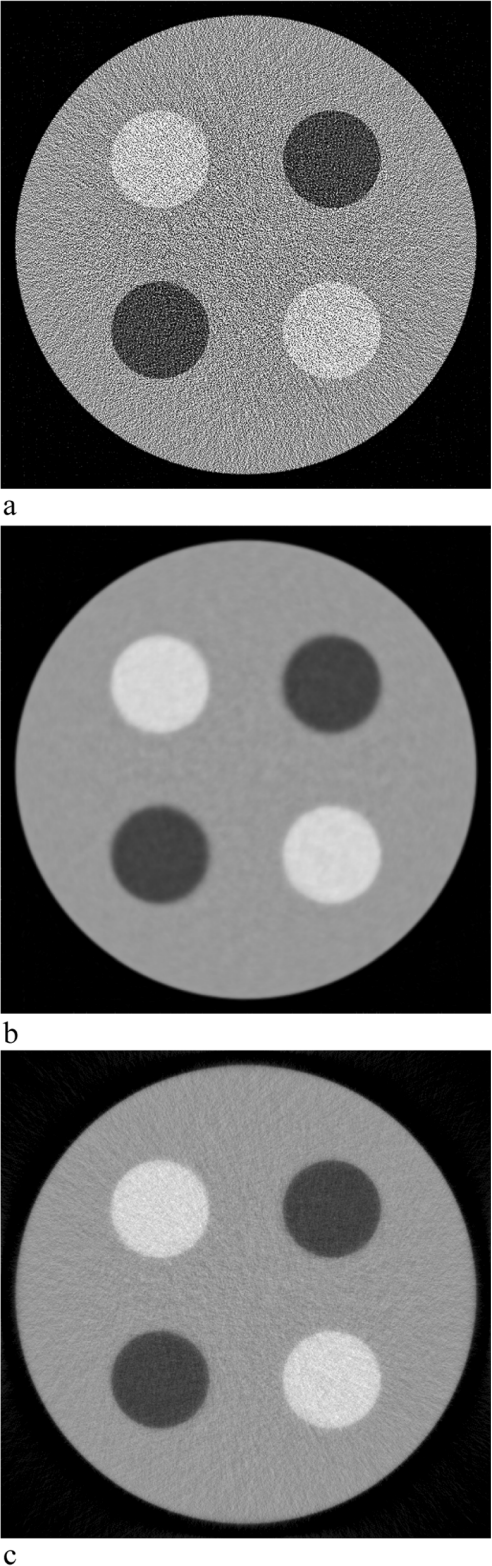
Larger image display for lower noise transmission data reconstructions. **a**: View of the upper-right image in Fig. 5 (EM-lookalike algorithm); **b**: View of the upper-right image in Fig. 6 (proposed algorithm); **c**: View of the upper-right image in Fig. 9 (POCS algorithm in ref. [12])

We point out that when large 512 × 512 images are displayed as small binned-down images, as in Figs. 5–10, image details are lost. At iteration 10,000, all algorithms are considered converged. We zoom in on the upper-right images in Figs. 5, 6, and 9 in Fig. 11. Here, one can better observe the differences between them. It is observed that the proposed Bayesian algorithms are effective in noise regularization, and stable as the iteration number increases.

The iterative POCS algorithm in our patient study provides better (yet noisier) spatial resolution than the proposed algorithm. The spatial resolution of an image reconstructed by the proposed iterative algorithms depends on the iteration number as well as the Bayesian penalty function. Usually, a larger iteration number gives a better spatial resolution, but a noisier reconstruction. The tradeoff between the spatial resolution and image noise is a main decision factor in selecting the iteration number. Suitable selection of the Bayesian penalty function, i.e., the constraints, plays an important role in the quality of the final reconstruction.

## Conclusions

Our proposed algorithms are inspired by Green’s OSL EM algorithm. The main novelty of this study is to propose a general methodology that extends EM-lookalike algorithms into MAP algorithms through a new multiplication factor (1*-βU*). We claim that our approach can be extended to any multiplicative updating reconstruction algorithm, where image non-negativity is built in. Thus, the proposed algorithms also have an intrinsic non-negativity constraint. The proposed algorithms are simple to implement, and they simultaneously optimize all constraints (instead of using POCS).

We implemented the POCS algorithm presented in ref. [12] for transmission tomography, and we utilized the TV norm and MSE to evaluate the reconstructions. We observed that our proposed simultaneous optimization algorithm outperforms the POCS algorithm proposed in ref. [12] for our experiments.

## Data Availability

Not applicable.

## Abbreviations

EM: Expectation maximization
FBP: Filtered backprojection
MAP: Maximum a posterior
ML: Maximum likelihood
MSE: Mean-squared-error
OSL: One-step-late
PET: Positron emission tomography
POCS: Projection onto convex sets
PWLS: Penalized weighted least-squares
SPECT: Single photon emission computed tomography
TV: Total variation
